# Female Sex is Associated With Increased Reported Injury Rates and Difficulties With Use of Orthopedic Surgical Instruments

**DOI:** 10.7759/cureus.14952

**Published:** 2021-05-11

**Authors:** Brianna Fram, Meghan E Bishop, Pedro Beredjiklian, Daniel Seigerman

**Affiliations:** 1 Orthopaedic Surgery, Thomas Jefferson University, Philadelphia, USA; 2 Sports Medicine, Rothman Orthopaedic Institute, New York, USA; 3 Division of Hand Surgery, Rothman Orthopaedic Institute, Philadelphia, USA; 4 Orthopaedic Surgery, Rothman Orthopaedic Institute, New York, USA

**Keywords:** hand injury, female, orthopedic surgery, surgery, ergonomics

## Abstract

Introduction: Orthopedic instrumentation is generally made as one-size-fits-all. The purpose of this study was to evaluate the effects of hand size and sex on ease of use and injury rates from orthopedic tools and surgical instruments.

Methods: An anonymous 21-item online survey was distributed to orthopedic trainees and attendings. Questions regarding demographics, physical symptoms and treatment, perceptions, and instrument-specific concerns were included. The analysis included statistics comparing responses based on sex, height, and glove size, with significance as p<0.05.

Results: There were 204 respondents: 119 female and 84 male. Male and female respondents differed significantly in height (mean difference 5.4 in, p<0.001) and glove size (median size 6.5 size for females, size 8 for males, p<0.001). While 69.8% of respondents reported physical discomfort or symptoms they attributed to their operating instruments, female surgeons were significantly more likely to endorse symptoms (87.3% female vs. 45.2% male, p<0.001). Of those reporting symptoms, 47.7% had undergone treatment, with no significant difference by surgeon sex (p=0.073). Female surgeons were significantly more likely than their male counterparts to have negative attitudes toward orthopedic surgical instruments and to report specific surgical instruments as difficult or uncomfortable to use.

Conclusion: Female orthopedic surgeons are more likely than their male counterparts to report physical symptoms attributed to orthopedic surgical instruments, to have negative attitudes toward instruments, and to identify a larger number of common instruments as difficult or uncomfortable to use. Further emphasis on ergonomic instrument design is needed to allow all orthopedic surgeons to operate as safely and effectively as possible.

## Introduction

As of 2016-2017, orthopedic surgery remains the medical specialty with the lowest proportion of female residents (14.0%) and female medical school faculty (17.8%) [1]. Further, it is a field with a wide variety of instrument types and implant sets, which are predominantly sold by for-profit companies that often consult with surgeons regarding implant design [2]. Data from 2013 showed that 50.1% of orthopedic surgeons reported a financial relationship with industry, with <80% of the value of reported industry income coming from royalties, licensing, and consulting, more than any other surgical sub-specialty [3]. While there is no published data on the gender breakdown of orthopedic surgeons who consult for industry, literature from a variety of medical specialties has shown male surgeons are more likely to have consulting relationships with industry and therefore may be more likely to consult on implant and hardware design than female surgeons [4]. This could lead to a potential bias of the ergonomics of these devices and their accompanying equipment for taller male surgeons with larger hands.

Based on these facts, we sought to evaluate the effects of surgeon hand size, height, and sex on ease of use and injury rates for orthopedic tools and surgical instruments. We did this using an anonymous internet survey of orthopedic trainees and practicing attendings. We attempted to identify risk factors for poor perceived ergonomics, as well as instrument-specific concerns amongst orthopedic surgeons. Our hypothesis was that shorter height, smaller hand size, and female gender would predispose surgeons to musculoskeletal symptoms attributed to and negative perceptions toward orthopedic surgical instruments.

## Materials and methods

**Figure 1:**
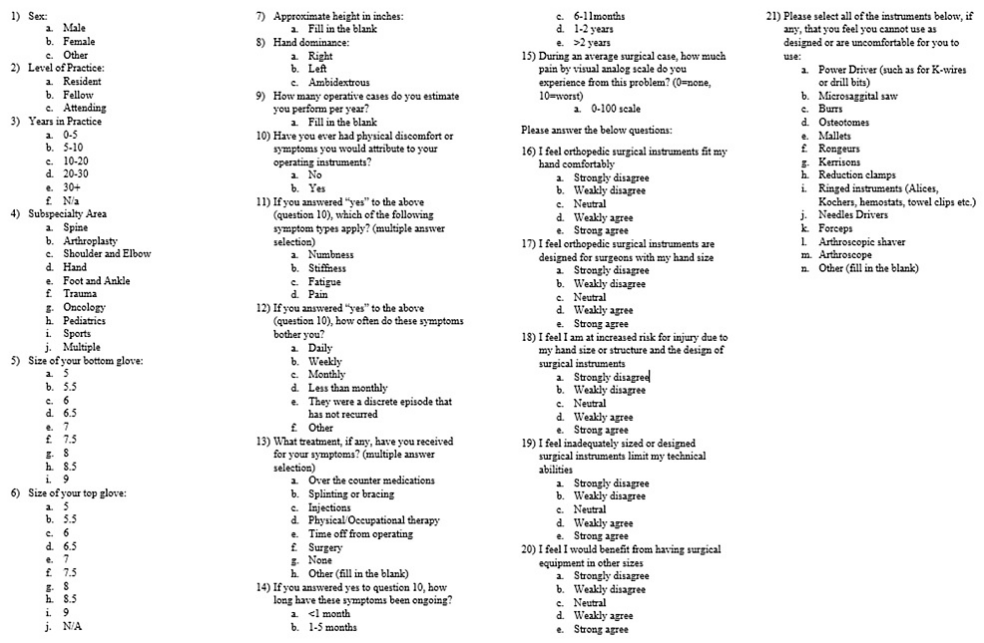
Survey questions Copy of survey questions that were sent to respondents.

We developed an anonymous 21-item online survey, with questions in the categories of demographics, physical symptoms and treatment, perceptions, and instrument-specific concerns. Surgeon attitudes toward surgical instruments were assessed by five questions, using a five-point scale of strongly disagree, weakly disagree, neutral, weakly agree, or strongly agree (Figure 1). This was distributed by internet link to a medium-sized orthopedic residency program via email (24 of 29 responded, 82.8%), to the alumni mailing list and active attending list of a large single-specialty orthopedic practice group via email (71 of 459 responded, 15.5%), and to a private Women in Orthopaedics Facebook group made up of female orthopedic residents, fellows, and attendings via posted link (110 of 1180 members responded, 9.3%). Survey results were exported to Microsoft Excel and analyzed using SPSS statistical software (IBM Corp, Armonk, NY; version 25). Descriptive statistics included means and standard deviations for normally distributed numeric variables, median for discrete variables, and distributions for categorical variables. Comparative analysis for normally distributed numeric variables was performed with Student’s t-test or ANOVA, and analysis of non-parametric numeric variables was performed with Mann-Whitney U test or Kruskal-Wallis H test, based on the number of categorical groups. Chi-square analysis was performed for comparison of categorical variables. Logistic regression was performed for comparison of continuous normally distributed numeric variables. Statistical significance was set at p=0.05. This study was exempt from IRB approval by our institution.

## Results

There were 204 respondents. Demographically, 119 (58.6%) were female, and 84 (41.4%) male. Regarding the level of training, 43 (21.2%) were residents, 10 (4.9%) fellows, and 151 (74.0%) attendings. The majority of respondents had been in practice for 0-5 years (86 [42.2%]). All orthopedic subspecialties were represented with hand (41 [20.2%]), sports (31 [15.8%]), and pediatrics (32 [15.8%]) the most common. The majority of respondents were right-hand dominant (179 [87.7%]). Respondents reported performing a mean of 363 operative cases per year (SD=200; Table 1).

**Table 1:** Demographics Survey respondent demographics by sex.

Level of training	Male	Female	All	P-value
Resident	18 (21.4%)	25 (21.0%)	43 (21.1%)	0.024
Fellow	0	10 (8.4%)	10 (4.9%)	
Attending	66 (78.6%)	84 (70.6%)	151 (74.0%)	
Years in practice				
N/A	3 (3.6%)	5 (4.2%)	8 (3.9%)	<0.001
0–5	22 (25.6%)	64 (53.8%)	86 (42.2%)	
5–10	7 (8.3%)	22 (18.5%)	29 (14.2%)	
10–20	16 (19.0%)	24 (20.2%)	40 (19.6%)	
20–30	18 (21.4%)	3 (2.5%)	22 (10.8%)	
30+	18 (21.4%)	1 (0.8%)	19 (9.3%)	
Subspecialty				
Arthroplasty	16 (19.0%)	6 (5.1%)	22 (10.8%)	0.001
Foot and ankle	3 (3.6%)	5 (4.2%)	8 (3.9%)	
Hand	19 (22.6%)	22 (18.6%)	41 (20.2%)	
Multiple	14 (16.7%)	12 (10.2%)	26 (12.8%)	
Oncology	1 (1.2%)	2 (1.7%)	3 (1.5%)	
Pediatrics	4 (4.8%)	28 (23.7%)	32 (15.8%)	
Shoulder and elbow	5 (6.0%)	4 (3.4%)	9 (4.4%)	
Spine	9 (10.7%)	6 (5.1%)	16 (7.9%)	
Sports	10 (11.9%)	22 (18.6%)	32 (15.8%)	
Trauma	3 (3.6%)	11 (9.3%)	14 (6.9%)	
Hand dominance				
Right	75 (89.3%)	103 (86.6%)	179 (87.7%)	0.524
Left	5 (6.0%)	12 (10.1%)	17 (8.3%)	
Ambidextrous	4 (4.8%)	4 (3.4%)	8 (3.9%)	
Cases per year	431 (217)	317 (173)	363 (200)	<0.001
Height (in)	70.8 (2.7)	65.3 (3.0)	67.6 (4.0)	<0.001

Overall, 141/202 (69.8%) of respondents reported physical discomfort or symptoms they attributed to their operating instruments. Fatigue and pain were the most common symptom types. Of those who reported symptoms, the most commonly reported frequency was weekly (34.3%), with only 12.6% reporting their symptoms had been a discrete episode that had not recurred. The median symptom duration was 1-2 years. Mean pain, by visual analog scale (VAS), reported during an average OR case was 13.6/100 (SD=14.5; Table 2). Of surgeons reporting symptoms, 47.7% had undergone treatment for them. This was most commonly in the form of over-the-counter medications (Table 3).

**Table 2:** Presence and classification of symptoms attributed to surgical instruments

Symptoms by gender	Male	Female	All	P-value
Physical symptoms				
Yes	38 (45.2%)	103 (87.3%)	141 (69.8%)	<0.001
No	46 (54.8%)	15 (12.7%)	61 (30.2%)	
Type of symptoms				
Numbness	12 (14.3%)	30 (25.2%)	42 (20.7%)	0.078
Stiffness	17 (20.2%)	37 (31.1%)	54 (26.6%)	0.107
Fatigue	31 (36.9%)	84 (70.6%)	115 (56.7%)	<0.001
Pain	30 (35.7%)	75 (63.0%)	105 (51.7%)	<0.001
Frequency of symptoms				
Daily	5 (13.2%)	10 (9.5%)	15 (10.5%)	0.743
Weekly	9 (23.7%)	40 (38.1%)	49 (34.3%)	
Monthly	10 (26.3%)	22 (21.0%)	32 (22.4%)	
Less than monthly	8 (21.1%)	18 (17.1%)	26 (18.2%)	
Discrete single episode	5 (13.2%)	13 (12.4%)	18 (12.6%)	
Duration of symptoms				
<1 month	10 (26.3%)	15 (14.7%)	25 (17.9%)	0.023
1–5 months	0	12 (11.8%)	12 (8.6%)	
6–11 months	0	5 (4.9%)	5 (3.6%)	
1–2 years	7 (18.4%)	30 (29.4%)	37 (26.4%)	
>2 years	21 (55.3%)	40 (39.2%)	61 (43.6%)	
Pain by VAS during average case	11.1 (13.5)	14.8 (14.9)	13.6 (14.5)	0.137

**Table 3:** Treatment of symptoms attributed to surgical instruments

Treatment by gender	Male	Female	All	P-value
Any treatment				
Yes	16 (35.6%)	55 (52.9%)	71 (47.7%)	0.073
No	29 (64.4%)	49 (47.1%)	78 (52.3%)	
OTC meds				
Yes	12 (33.3%)	43 (41.0%)	55 (39.0%)	0.553
Splinting				
Yes	1 (2.8%)	30 (28.6%)	31 (22.0%)	0.001
Injection(s)				
Yes	2 (5.6%)	17 (16.2%)	19 (13.5%)	0.157
PT/OT				
Yes	1 (2.8%)	7 (6.7%)	8 (5.7%)	0.68
Time off from OR				
Yes	3 (8.3%)	5 (4.8%)	8 (5.7%)	0.424
Surgery				
Yes	1 (2.9%)	9 (8.7%)	10 (7.2%)	0.451
Other				
Yes	4 (8.9%)	7 (6.7%)	11 (7.3%)	0.76

**Figure 2:**
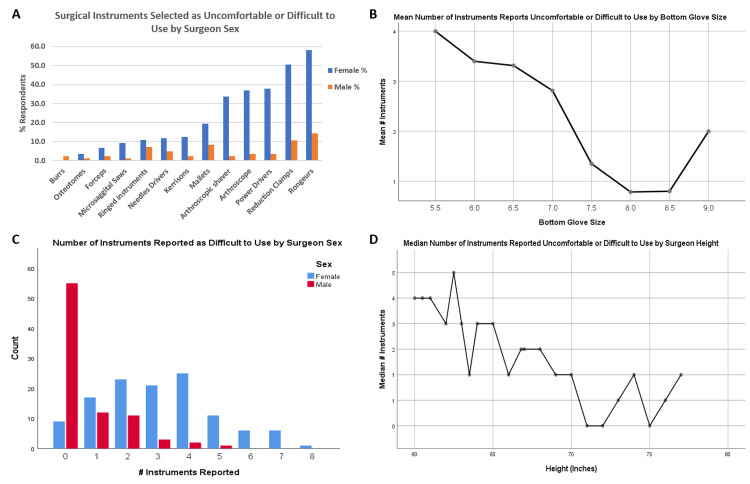
Difficult instruments graph Surgical instruments selected as uncomfortable or difficult to use by surgeon characteristics: (A) uncomfortable or difficult to use surgical instruments by the percentage of respondents of each sex; (B) mean number of instruments, out of 14 possible instruments, based on surgeon bottom glove size; (C) distribution of the number of instruments reported uncomfortable or difficult to use, by surgeon sex; (D) the median number of instruments reported uncomfortable or difficult to use, by surgeon height in inches.

Respondents reported a median of two surgical instruments as uncomfortable or difficult to use. The most commonly reported instruments were rongeurs (81/204, 39.7%), reduction clamps (69/204, 33.8%), and power drivers (48/204, 23.5%; Figure 2).

Sex

**Figure 3:**
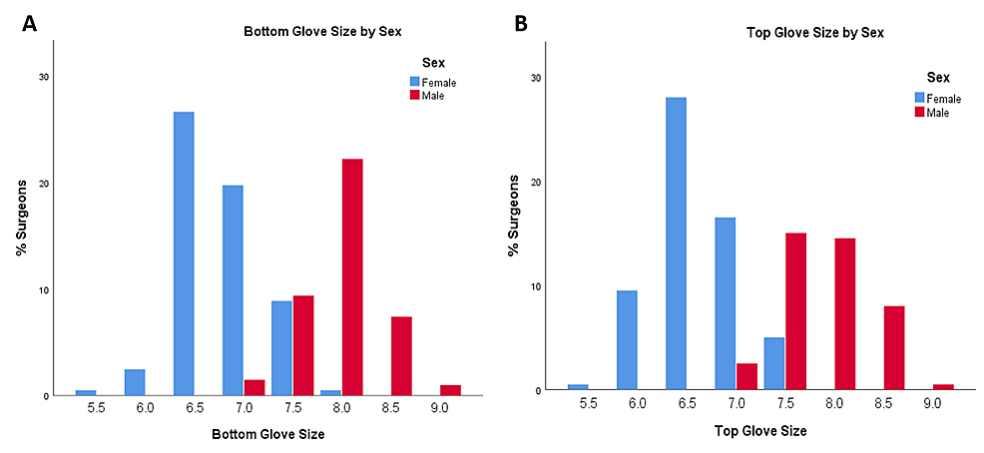
Glove size charts Graphs of Glove Size by Surgeon Sex. (A) Bottom surgical glove size by surgeon sex and (B) top surgical glove size by surgeon sex.

**Figure 4:**
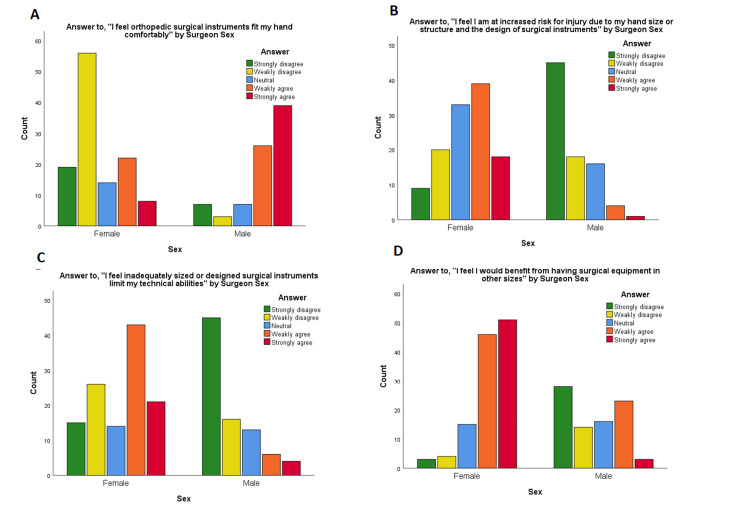
Attitudes by sex Attitudes toward orthopedic surgical instruments by surgeon sex: (A) Answers to, “I feel orthopedic surgical instruments fit my hand comfortably” by surgeon sex; (B) answers to, “I feel I am at increased risk for injury due to my hand size or structure and the design of surgical instruments” by surgeon sex; (C) answers to, “I feel inadequately sized or designed surgical instruments limit my technical abilities” by surgeon sex; (D) answers to, “I feel I would benefit from having surgical equipment in other sizes” by surgeon sex.

Male and female respondents did not differ significantly by the level of training or hand dominance. They did differ significantly for years in practice (with men more likely to have been in practice longer), orthopedic subspecialty, reported operative cases per year (with men more likely to report more cases/year), and reported height (all p≤0.001, Table 1). Median bottom and top glove sizes for females were both 6.5, and for males, both were 8.0 (p<0.001; Figure 3). Female surgeons were significantly more likely than male surgeons to report physical symptoms they attributed to surgical instruments (87.3% vs. 45.2%, respectively, p<0.001; Table 2). There was no significant difference by sex in the likelihood a surgeon had undergone treatment for these symptoms, though there was a trend toward female surgeons having treatment at higher rates (35.6% males vs. 52.9% females, p=0.073; Table 3). Female surgeons were significantly more likely than their male counterparts to have negative attitudes toward orthopedic surgical instruments (p<0.001 for all five questions; Table 4, Figure 4). Female surgeons were also significantly more likely than male surgeons to report surgical instruments as difficult or uncomfortable to use, with a median of 3 instruments out of 14 options vs. 0 instruments for male surgeons (p<0.001; Figure 2).

**Table 4:** Attitudes toward orthopedic surgical instruments

	All respondents						Significance testing by characteristic			
	Strongly disagree	Weakly disagree	Neutral	Weakly agree	Strongly agree	Median response	Gender	Bottom glove size	Height	Hand dominance
I feel orthopedic surgical instruments fit my hand comfortably	26 (12.9%)	59 (29.2%)	21 (10.4%)	49 (24.3%)	47 (23.3%)	Neutral	P<0.001	P<0.001	P<0.001	0.84
I feel orthopedic surgical instruments are designed for surgeons with my hand size	49 (24.0%)	49 (24.0%)	32 (15.7%)	41 (20.1%)	33 (16.2%)	Neutral	P<0.001	P<0.001	P<0.001	0.767
I feel I am at increased risk for injury due to my hand size or structure and the design of surgical instruments	54 (26.5%)	38 (18.6%)	50 (24.5%)	43 (21.1%)	19 (9.3%)	Neutral	P<0.001	P<0.001	P<0.001	0.499
I feel inadequately sized or designed surgical instruments limit my technical abilities	60 (29.4%)	43 (21.1%)	27 (13.2%)	49 (24.0%)	25 (12.3%)	Weakly disagree	P<0.001	P<0.001	P<0.001	0.441
I feel I would benefit from having surgical equipment in other sizes	31 (15.2%)	18 (8.8%)	32 (15.7%)	69 (33.8%)	54 (26.5%)	Weakly Agree	P<0.001	P<0.001	P<0.001	0.496

Height

**Figure 5:**
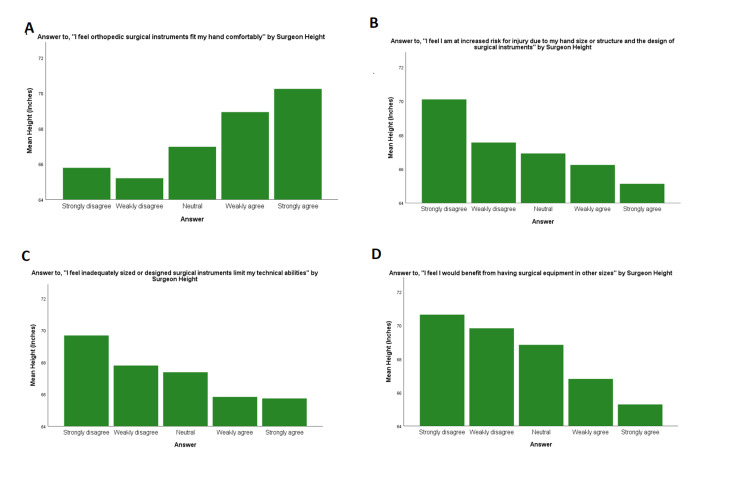
Attitudes by height Attitudes toward orthopedic surgical instruments by surgeon height.

When each sex was analyzed separately, height was not significantly associated with instrument-related symptoms (p=0.151 for male surgeons, p=0.448 for female surgeons). On subgroup analysis by sex, shorter height was significantly correlated with negative attitudes for 3/5 questions within female surgeons (p=0.015 for hand comfort, p=0.005 for instrument design by hand size, p=0.286 for risk of injury, p=0.474 for technical abilities, and p=0.024 for equipment in other sizes) and 0/5 within male surgeons (p=0.763 for hand comfort, p=0.875 for instrument design by hand size, p=0.571 for risk for injury, p=0.468 for technical abilities, and p=0.929 for equipment in other sizes; Figure 5). Decreased height was significantly correlated with an increased number of surgical instruments identified as difficult or uncomfortable to use (p<0.001; Figure 1). When each sex was analyzed separately, this trended toward but did not meet significance for female surgeons (p=0.078) and did not meet significance for male surgeons (p=0.734).

Bottom glove size

When each sex was analyzed separately, bottom glove size was not significantly associated with instrument-related symptoms (p=0.947 for male surgeons, p=0.786 for female surgeons). On subgroup analysis by sex, smaller bottom glove size was significantly correlated with negative attitudes for 4/5 questions within female surgeons (p=0.001 for hand comfort, p<0.001 for instrument design by hand size, p=0.158 for risk of injury, p=0.032 for technical abilities, and p<0.001 for equipment in other sizes) and 1/5 within male surgeons (p=0.365 for hand comfort, p=0.302 for instrument design by hand size, p=0.179 for risk for injury, p=0.026 for technical abilities, and p=0.351 for equipment in other sizes). When each sex was analyzed separately, smaller bottom glove size was not significantly correlated with an increased number of surgical instruments identified as difficult or uncomfortable to use for female (p=0.183) or male surgeons (p=0.155).

Hand dominance

Hand dominance was not significantly correlated with the likelihood of reporting surgical instrument-related symptoms (p=0.469), negative attitudes toward orthopedic surgical instruments (p>0.05 for all five questions; Table 4), or with an increased number of surgical instruments identified as difficult or uncomfortable to use (p=0.352).

## Discussion

Orthopedic instrumentation is generally made as one-size-fits-all without consideration of different size and strength characteristics in the surgeons' users. We found that female orthopedic surgeons were significantly more likely than male orthopedic surgeons to report physical discomfort or symptoms attributable to surgical instruments, to have negative attitudes toward orthopedic surgical instrument design, and to identify a larger number of common instruments as difficult or uncomfortable to use. Further, we identified specific instruments that a high percentage of female respondents found difficult to use, including rongeurs, reduction clamps, power drivers, and arthroscopic equipment such as cameras and shavers. Shorter surgeon height and smaller bottom glove size also independently correlated with a more negative attitude toward orthopedic surgical instruments within the subset of female surgeons. Smaller bottom glove size and shorter surgeon height did not correlate with a higher likelihood of physical symptoms or a larger reported number of difficult to use instruments after we controlled for surgeon gender, though shorter height did trend toward significance for a number of reported instruments within female surgeons. We did not find significant effects based on hand dominance.

Smaller hand size has been correlated with increased difficulty using various types of surgical instruments in surveys among general surgeons [5,6]. Additionally, female surgeons have been shown to have more discomfort and seek more medical treatment for hand conditions than their male counterparts with the same glove size [7]. Our results within the orthopedic community also identified effects of sex, hand size, and height on surgeon-reported physical symptoms, instrument perceptions, and instrument-specific concerns. When controlling for confounding factors, gender alone was positively correlated with all three domains.

There is a growing body of evidence that orthopedic surgery is lagging behind other specialties in gender diversity, both by percentage and rate of change [1,8]. Orthopedics had the second-lowest percentage increase in female residents from 2006 to 2015, rising by only 3.5% (from 10.9% to 14.4%) [8]. The root of this is certainly multifactorial, with recent literature indicating perceived bias against pregnancy and parenthood among female orthopedic residents, concerns about work-life balance and physical demands, and lack of available mentors, among many other proposed contributors [9,10]. Based on our results, perceptions about instrument design may be another facet of this. When those within a field perceive that key aspects of their profession are not designed to accommodate them, those attitudes can be permeative and possibly even perceptible to impressionable medical students. It is important to make the environment of orthopedics suitable for both male and female surgeons. The ultimate advantage of having diversity among orthopedic surgeons is to provide excellent care to our diversified pool of orthopedic patients. An important aspect of this would be to have equipment available to suit all surgeons irrespective of strength, height, glove size, and gender.

Additionally, nearly half of all male surgeons in our cohort reported physical discomfort attributed to surgical instruments. The need to address workplace ergonomic concerns is not unique to orthopedics. The nursing community has identified difficulty in performing necessary tasks such as moving and turning patients as a potential occupational hazard. Lee et al. reported on musculoskeletal pain among critical care nurses when performing typical occupational duties. They found that greater availability of lifts and other associated equipment was associated with less musculoskeletal pain among participants [11]. This is an example of how appropriate equipment improves a healthcare workers’ ability to perform occupational duties. There could potentially be similar results if appropriately sized and weighted orthopedic instrumentation were available for all surgeons.

Existing literature on ergonomic surgical instrument design suggests precision tools need to be manufactured in multiple sizes to allow ergonomic use through the range of hand sizes [12]. Further, the limited research we identified on the gender effects of hand mechanics indicates significant differences in force generation between male and female hands [13]. Multiple fields of surgery, including adult reconstructive surgery and spine surgery within orthopedics, have begun to acknowledge the toll poor ergonomics and work-related injuries can take on surgeons’ careers and their overall health and well-being [14-19]. Despite the fact we did not find significant correlations between surgeon glove size and surgeon height with the likelihood of physical symptoms, we believe that the ergonomic use of surgical instruments is multifactorial and may include factors such as grip strength and hand structure and mechanics. Further study is needed to explore these elements in order to move toward designing more ergonomically sound instrumentation for all hand types and genders. We feel the field of orthopedics would benefit greatly from a design focus on instruments for surgeons of diverse heights, handednesses, hand sizes, and structures, to maximize the productivity and longevity of our surgeons.

Study limitations

As in all surveys, the design of this study was limited by question design and selection bias. This includes voluntary response bias, where respondents with strong feelings about the topic of interest are more likely to respond. It was difficult to tell how many members of the Facebook group were active or regularly checking the posting page. However, our email response rate was only 95/488 (19.5%). Further, the majority of male and female respondents came from different sources (practicing attendings from a single-specialty practice group and alumni of its residency program for males, an invite-only female orthopedic surgeon social media group for females); these sources may have had different cultural norms or biases. Given the small proportion of female surgeons in most orthopedic societies, we did feel surveying a largely female group was the only way to obtain an adequate sample size. There were significant differences in multiple demographic characteristics between the male and female respondents. It is difficult to know how these differences compare to the overall population of orthopedic surgeons and how they may have affected survey responses. However, using these sources we were able to collect data from a variety of surgeons with varying levels of experience and specialties, strengthening the generalizability of our data across the field of orthopedics.

## Conclusions

We found higher rates of self-reported physical symptoms attributed to surgical instrument use among female orthopedic surgeons than male orthopedic surgeons. Further, a higher number of female surgeons felt surgical instruments did not fit their hands comfortably, were not designed for surgeons of their hand size or structure, put them at increased risk of injury, limited their technical abilities, and should be available in more sizes. Females surgeons identified more difficulties handling instruments than their male counterparts. Further study of the effects of hand size and gender on risk for injury from orthopedic instruments, attitudes toward these instruments, and specific problematic instruments can lead to awareness of these issues and design of surgical tools that allow all orthopedic surgeons to operate as safely and effectively as possible.
